# Surgical treatment results for dupuytren's disease

**DOI:** 10.1590/1413-785220172503164827

**Published:** 2017

**Authors:** Serkan Aykut, Mehmet Baydar, Abdul Fettah Büyük, İbrahim Avşin Öztürk, Erdem Özden, Kahraman Öztürk

**Affiliations:** 1 Metin Sabancı Baltalimanı Bone Diseases Training and Research Hospital, Department of Hand Surgery, Istanbul, Turkey; 2 Haseki Training and Research Hospital, Department of Orthopedics and Traumatology, Istanbul, Turkey; 3 Toros Public Hospital, Department of Orthopedics and Traumatology, Mersin, Turkey

**Keywords:** Dupuytren contracture/surgery, Dupuytren contracture/therapy, Fasciotomy

## Abstract

**OBJECTIVE:**

To present the results of our cases of Dupuytren's disease treated with regional selective fasciectomy in light of the literature.

**METHODS:**

Patients diagnosed with Dupuytren's contracture and surgically treated with regional selective fasciectomy at our institution with adequate follow-up data were included in the study. All patients were routinely followed after surgery to assess results and complications. QuickDASH scoring was used to evaluate the patients and recurrences and complications were recorded.

**RESULTS:**

Twenty-one hands of 19 patients (13 males, 6 females) who underwent surgery and received adequate follow-up were retrospectively evaluated. Mean patient age was 65.8 (range: 41 to 86) and the mean follow-up period was 48.2 months (range: 24 to 86). Fourteen (66.6%) hands had excellent results, five (23%) hands had good results and two (9.4%) had fair results. The mean QuickDASH score for the patients at the final follow-up was 6.58 (range: 0 to 20.4).

**CONCLUSION:**

Our study results demonstrated that regional selective fasciectomy is a reliable and efficient method to treat Dupuytren's disease with low rates of complications and recurrence and the technique can be considered the gold standard. ***Level of Evidence IV, Case Series.***

## RESUMO

**OBJETIVO:**

Apresentar os resultados de nossos casos de doença de Dupuytren tratados com fasciotomia seletiva regional, à luz da literatura.

**MÉTODOS:**

Os pacientes diagnosticados com contratura de Dupuytren e tratados cirurgicamente com fasciotomia seletiva regional em nossa instituição que tinham dados de acompanhamento adequados foram incluídos no estudo. Todos os pacientes foram rotineiramente acompanhados após a cirurgia para avaliação dos resultados e das complicações. Foi utilizada a pontuação QuickDASH na avaliação dos pacientes e as recorrências e complicações foram registradas.

**RESULTADOS:**

Foram avaliadas retrospectivamente vinte e uma mãos de 19 pacientes (13 homens, 6 mulheres) submetidos à cirurgia e acompanhados adequadamente. A média de idade dos pacientes foi de 65,8 (intervalo: 41 a 86) e o período médio de seguimento foi 48,2 meses (intervalo: 24 a 86). Quatorze (66,6%) mãos tiveram excelentes resultados, enquanto cinco (23%) mãos tiveram bons e duas (9,4%) tiveram resultados moderados. A pontuação média no QuickDASH dos pacientes no seguimento final foi de 6,58 (intervalo: 0 a 20,4).

**CONCLUSÃO:**

Os resultados do nosso estudo demonstraram que a fasciotomia seletiva regional é um método confiável e eficiente, com baixas taxas de complicação e recorrência no tratamento da doença de Dupuytren e a técnica pode ser considerada o padrão-ouro. ***Nível de Evidência IV, Série de Casos.***

## INTRODUCTION

Dupuytren's disease is a benign fibroproliferative disorder of the palmar and digital fascia. The disease usually starts with a palpable nodule (the Dupuytren nodule) in the palm and may cause flexion contracture in the joints and functional impairment as it progresses.^1^ ^-^ ^5^ The etiology of the disease remains unclear. However, male sex, advanced age, occupation, trauma, alcohol use, diabetes, smoking and epilepsy are known risk factors.^6^ ^-^ ^8^ Autosomal dominant inheritance with varying penetrance has been reported in several studies and the disorder has been confirmed in positive family histories.^4^ ^,^ ^9^ ^,^ ^10^

Treatment options can be categorized under four main sections; conservative approaches, collagenase injections, needle aponeurotomy and fasciectomy.^3^ Fixed-flexion contractures are usually treated with surgical methods. Surgical management is recommended for cases with contracture in the PIP joint or contracture over 30 degrees in the metacarpophalangeal joint, with the limited palmar fasciectomy method the most popular and recognized option.^11^ ^,^ ^12^

This study presents the results in our cases who received surgical treatment for Dupuytren's disease, in light of the literature. 

## PATIENTS AND METHODS

Patients diagnosed with Dupuytren's contracture and surgically treated with regional selective fasciectomy at our institution between May 2006 and May 2014 who had adequate follow-up data were included in the study. All patients signed a free and informed consent form. Patients were staged according to system by Khan et al.^13^ (Table 1) In addition, smoking habits, alcohol use, regular use of medications and accompanying chronic diseases were noted for each patient. Since this study is retrospective in nature, institutional review board approval was not necessary.

**Table 1:** Clinical staging of the patients.

Staging	Clinical characteristics
Stage 1	Thickened nodule and band in the palmar aponeurosis; may have associated skin abnormalities
Stage 2	Limitation of finger extension in addition to Stage 1
Stage 3	Presence of flexion contracture in addition to Stage 2

**Figure 1:**
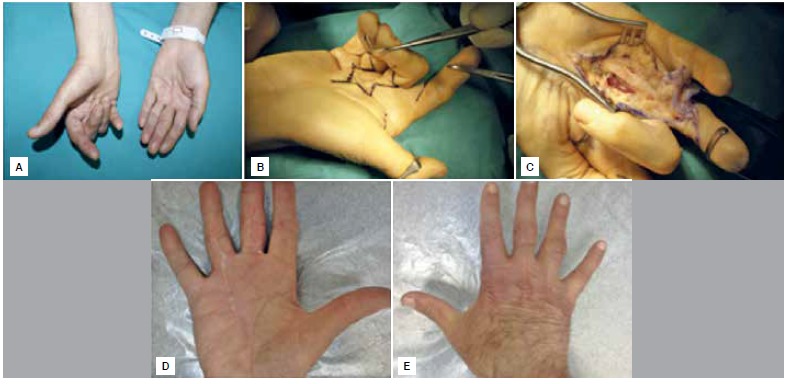
(A) Preoperative image of a patient with involvement in the 3^rd^ and 4^th^ digit of the right hand. (B) Planning for zigzagplasty. (C) Appearance of 3^rd^ digit after removal of diseased tissue. (D, E) Functional outcome at 24^th^ months post-procedure.

All surgeries were carried out using an infraclavicular block with the application of a pneumatic tourniquet. Patients were given prophylactic first-generation cephalosporin for the 24 hours before and after surgery. Zigzagplasty extending straight toward the proximal or direct incision with multiple z-plasties was employed for the surgical incision. (Figure 1A-C) Regional fasciectomy (excision of the involved fascia) was performed in all patients and all surgeries were performed under magnification. After release of the tourniquet, the site was checked for bleeding and an aspiration drain was used. Skin grafting was required for wound closure in one patient and primary closure was performed in the others.

A short arm splint was applied postoperatively to maintain the hand and fingers in extension. After the edema subsided, the splint was removed and rehabilitation initiated. All patients continued to use the extension splint at night for three months.

All patients were routinely followed after surgery to assess results and complications. QuickDASH scoring was used for patient evaluation, challenges during functional recovery and daily activities were investigated and recurrences and complications were recorded. (Figure 1D, E)

We grouped our results into four sections, as suggested by Khan et al.^13^ According to this classification, full movement/function and no recurrence was considered 'excellent,' mild loss of flexion-extension in fingers with minor impact on function was considered 'good,' loss of function with joint stiffness, recurrence and limitation in daily activities was considered 'fair,' and severe loss of function and failure to recover after the first contracture was considered 'poor.' (Table 2)

**Table 2:** Classification of patient outcomes.

Results	Movement/Function/Recurrence
Excellent	Full movement and function, no recurrence
Good	Mild loss of flexion-extension in fingers with minor impact on function
Fair	Loss of function with joint stiffness, recurrence, limitation in daily activities
Poor	Failed to recover, severe loss of function

## RESULTS

Twenty-one hands in 19 patients (13 males, 6 females) who underwent surgery and had adequate follow-up were retrospectively evaluated. Mean patient age was 65.8 (range: 41 to 86) and mean follow-up period was 48.2 months (range: 24 to 86). Fourteen (66.6%) hands had excellent results, five (23%) hands had good results and two (9.4%) had fair results. Mean QuickDASH score for patients at the final follow-up was 6.58 (range: 0 to 20.4). (Table 3)

**Table 3:** Our results.

Treatment outcome	Point score	%
Excellent	14.0	66.6
Good	5.0	23.0
Fair	2.0	9.5

Bilateral involvement was observed in two (10.5%) patients. Four other patients had Dupuytren nodules in the other hand (21%). All (100%) patients had either contracture of the finger or flexion contracture over 30 degrees, constituting severe involvement (Stage 3). The second digit was involved in three (14.2%) cases, the third digit in six cases (28.5%), the fourth digit in 13 cases (61%) and the fifth digit in 12 (57%) cases.

Six patients were regular smokers, three were regular drinkers and one patient used barbiturates for epilepsy. Two patients were diagnosed with diabetes.

In one patient, the digital artery at the radial side of the fifth digit was accidentally cut during surgery. Primary repair was performed in this patient and no circulation problems were observed in the follow-up examinations. Two other patients complained of numbness in their fingers and two patients experienced recurrences.

## DISCUSSION

Several methods with varying rates of success, complication and recurrence have been reported in the literature to manage Dupuytren's disease.^3^ A general review of these methods will lead to better recovery, clinical outcome, morbidity and recurrence rates in cases treated with aggressive tissue dissection.^3^ Regional selective fasciectomy remains the gold standard in surgical treatment of Dupuytren's disease. The goal of the technique is to remove the macroscopically affected diseased fascia. Only regional selective fasciectomy was performed in our study and 90% of the patients had excellent and good results; excluding the two cases which developed recurrence. 

Duthie and Chesney^14^ performed percutaneous needle fasciectomy on 82 patients and followed them for 10 years. These authors observed a recurrence rate of 66%. In their series of 100 patients, Tonkin et al.^15^ compared dermofasciectomy with selective fasciectomy and reported that the recurrence rate was lower in patients who had undergone dermofasciectomy. Dermofasciectomy is still a valid treatment option in patients with recurrence or extensive skin involvement.^3^ Although fasciectomy and selective fasciectomy are similar in terms of functionality and recurrence rates, complication and morbidity rates are strikingly higher with radical fasciectomy.^16^ Khan et al.^13^ employed regional fasciectomy in 27 of their 30 patients and reported excellent and good results in 97% of the patients after five years of follow-up. Özkaya et al.^4^ retrospectively evaluated patients who underwent partial selective fasciectomy over a 10-year period and observed complications in 16.6% of the patients, but no recurrence. Ribak et al.^17^ compared regional selective fasciectomy and percutaneous needle fasciectomy and found no difference in terms of functionality between these techniques. These authors reported less total loss of passive extension in open selective fasciectomy.

## CONCLUSION

In conclusion, selective fasciectomy is an effective technique to treat Dupuytren's disease. Key factors for higher rates of success and lower rates of complication and recurrence are a good command of anatomy and extreme attention during surgery, as well as efficient rehabilitation in the postoperative period.
